# The Tuberculosis Problem in Texas

**Published:** 1907-07

**Authors:** 


### EDITORIAL DEPARTMENT.

### THE TUBERCULOSIS PROBLEM IN TEXAS.

What to do with the hordes of consumptives that flock to Texas every year on the approach of winter is a problem difficult of solution. The State is literally overrun with them; that is, the larger cities, and, as the vast majority of them are moneyless, they are worse than mendicants; they are paupers for the most part, and as there is not, and can not be hospital accommodations for them, they live in the streets and parks and are dependent of charity for food. They are thus a menace to the public health; and in San Antonio—the most famous "health resort"—and the favored Mecca to which they journey, the resident population has already been infected, and the mortality reports now embrace deaths amongst old settlers. In San Antonio the cost of maintenance of the city (charity) hospital is the largest item of the city's expense, and the mayor, in desperation, and holding that it would be the lesser of the two evils, and that "humanity to the individual is inhumanity to the general public," caused the consumptive ward of the city hospital to be emptied; the consumptives, all who could walk, were turned into the streets. Of course, there was a howl, but what else could be done? I don't know.

It seems to me that this is the worst thing that could have been done, laying aside the sentimental aspect of the case; for, at large, they scatter the infection everywhere,—while having no chance at all for recovery. It is true that, crowded together in a ward thirty by eighty feet, they had little chance, God knows; but on the streets they have even less.

In this connection, it is suggested that the philanthropic people of San Antonio, and of other cities similarly afflicted, together with the united charities organization establish camps in the woods, somewhere, where these unfortunates can be sheltered and fed, until the State comes to their relief, which it must do, at next Legislature, two years off.

Meantime, let the State Health Officer and the Governor do what lies in their power to *cut off the supply*, stop the stream of pauper consumptives that is constantly pouring into the State;—deal with causes, and not with effect. An irrational policy seems to characterize the civilization of today. We deal with effects and ignore causes. It is entirely possible and practicable in this instance to strike effectively at the cause, the source, and arrest the inflow, for it is utterly impossible to handle the incoming hordes; to deal with them after they arrive.

In June, 1902, Attorney General Knox ruled that "consumption is a contagious disease dangerous to the public health," and under this ruling foreign immigrants afflicted with the disease are excluded and sent back.

The laws of Texas prohibit the importation into Texas of contagious and infectious diseases, and it is the intention of the Governor and the State Health Officer to invoke this law, and to notify the railroad and other transportation companies that it will be rigidly enforced.

I append the following from the Revised Civil Statutes of Texas, Title XCII:

Article 4324. . Whenever the Governor has reason to believe that the State of Texas is threatened at any point or place on the coast, border or elsewhere within the State with the introduction or dissemination of yellow fever contagion, or any other infectious and contagious disease, that can and should, in the opinion of the State Health Officer, be guarded against by State quarantine, he shall, by proclamation, immediately declare said quarantine against any and all such places, and direct the State Health Officer to promptly establish and enforce the restrictions and conditions imposed and indicated by said quarantine proclamation, and when from any cause the Governor can not act, and the exigencies of the threatened danger require immediate action, the State Health Officer is empowered to declare quarantine as prescribed in this article, and maintain same until the Governor shall officially take action as he may see proper.

And the following from the Penal Code, Chapter 6:

Article 478a. Any person who shall knowingly and wilfully violate any regulation of quarantine established by the Governor, the State Health Officer, or the health officer of any county or city of this State, shall be guilty of a misdemeanor, and upon conviction thereof shall be punished by fine of not less than twenty nor more than one thousand dollars.

Article 478b. If any conductor or person in charge of any train, ship, steamboat, or any other kind of common carriers, shall knowingly and wilfully bring into this State any person or thing contrary to the quarantine regulations as proclaimed by the Governor or State Health Officer, such conductor or person so knowingly and wilfully offending shall be guilty of a misdemeanor and upon conviction thereof shall be fined in any sum not to exceed five hundred dollars.

Quarantine, as it is generally understood—i. e., detention at the

State border or elsewhere for a definite or indefinite period—is by no means to be thought of. Neither the Governor, nor the State Health Officer contemplates such steps. In this particular, Dr. Brumby has been misunderstood, misquoted, misrepresented, and has come in for a liberal share of censure by the unthinking. The State, by no means, contemplates taking consumptives off of trains and detaining them in quarantine. To do so would involve an expense not contemplated by the law now provided for, and unless the supply of consumptive immigrants be cut off, such camps would be nothing less than permanent consumption hospitals. I say nothing of other difficulties; they suggest themselves. The inspector (inspectors must be provided and paid) must be sure of his diagnosis before detaining any person. He can not make a microscopic examination "while you wait," and taking off the wrong man might entail serious consequences to more persons than the invalid.

Texas will not, therefore, and very properly, undertake a quarantine against consumptives in the sense that quarantine against other diseases—such as run a short course, is understood; *consumption is not a quarantinable disease,* but railroad and other transportation companies will be put upon notice by proclamation that they will be prosecuted under the provisions of the law above quoted, if, after notice, they bring into the State a case of tuberculosis, and that's all there is, of the much discussed and cussed "Brumbyan Theory."

IN PURSUANCE OF THE ABOVE, our physicians endeavored to secure the passage of a bill at the last Legislature for a State sanitarium for indigent consumptives. It contemplated a large camp, at some suitable point, and asked for $150,000. This bill passed the House, but met opposition in the Senate, where it was argued that it would be folly to undertake to care for the outpourings of pauper consumptives from the whole United States, and that such camp would be kept full, all the time, not of Texas people, but of those who come here from other States. The bill died on the calendar.

The answer to that argument, by friends of the bill, who, of course, were instructed by the physicians, is, that we are not in the charity business, solely, nor do we propose to go into it; that such sanitarium is not to be created for the benefit of charity consumptive patients, only incidentally, but that they may be "corralled"—cared for till they recover or die, in order that the general public *may be protected from their infection.*

It is hoped and believed that by next session the physicians of the State will have educated the people and the legislators, and enlightened the public on the necessity of such law, and that a provision of the kind will be made.

Meantime, what are we going to do with those already in the State?

